# Using Late C-reactive Protein Measurement in Transient Tachypnea of the Newborn to Predict Bacteremia and Reduce Blood Sampling Frequency

**DOI:** 10.7759/cureus.87808

**Published:** 2025-07-13

**Authors:** Hsien-Kuan Liu, Ming-Chun Yang, Wan-Chun Lin, San-Nan Yang, Teck-Jin Tan, Yung-Ning Yang

**Affiliations:** 1 Pediatrics, E-Da Hospital, I-Shou University, Kaohsiung, TWN; 2 Internal Medicine, Yuan's General Hospital, Kaohsiung, TWN; 3 Pediatrics, Pingtung Christian Hospital, Pingtung, TWN

**Keywords:** blood draw time, c-reactive protein (crp), neonatal sepsis, pediatric nursing, transient tachypnea of the newborn

## Abstract

Background

Frequent blood draws in neonates pose significant challenges, including procedural pain, risk of anemia, and increased healthcare burden. Hospitalized neonates with respiratory distress often undergo multiple blood tests to rule out bacterial infections, despite the low incidence of neonatal bacteremia. C-reactive protein (CRP) levels rise 10-12 hours post-infection, suggesting that early testing may not be optimal for predicting bacteremia. This study evaluates whether delaying the initial blood test and relying on a single blood draw can effectively predict neonatal bacteremia, aiming to reduce unnecessary phlebotomy and improve clinical efficiency.

Methods

This retrospective study included neonates diagnosed with transient tachypnea of the newborn (TTN) at E-Da Hospital from January 2021 to June 2022. Cases with congenital anomalies, unstable vital signs, or maternal infections were excluded. Data on neonatal and maternal characteristics, laboratory findings, and blood culture results were analyzed. Logistic regression, random forest analysis, and penalized regression were used to determine predictors of neonatal bacteremia, with a focus on the timing of CRP measurements.

Results

A total of 406 neonates were included, with 10 cases (2.46%) of positive blood cultures. The mean gestational age was 37.8±2.38 weeks, and the average birth weight was 2935.88±600.08 g. The first CRP (measured at admission) was 0.41±2.67 mg/L, while the second CRP (measured 12-24 hours later) was 4.9±8.13 mg/L. Logistic regression identified the second CRP as the only independent predictor of bacteremia (adjusted odds ratio 1.09; 95% CI 1.03-1.15; p<0.05). In the two-variable random forest model, the second CRP had the highest importance score (0.7524) compared to the first CRP (0.2476). In the penalized regression model, the second CRP exhibited a higher standardized coefficient (+1.48) compared to the first CRP (+0.74), suggesting greater predictive importance after penalization. In the multivariable model incorporating gestational age, birth weight, white blood cell (WBC) count, and premature rupture of membranes (PROM), the late CRP remained the most important predictor (importance score 0.2702), followed by WBC count and birth weight. Mode of delivery and PROM had minimal predictive value.

Conclusion

Delaying the initial blood test and relying on a single blood draw post-birth can effectively predict bacteremia in neonates with TTN, reducing unnecessary phlebotomy and associated risks. This strategy minimizes neonatal pain, prevents iatrogenic anemia, and improves healthcare efficiency without compromising patient safety.

## Introduction

Neonatal sepsis is challenging to diagnose, as most cases initially lack specific symptoms. Neonates presenting with respiratory distress, fever, or decreased activity are often hospitalized for comprehensive blood testing and empirical antibiotic treatment [1,2]. However, there is no reliable marker to predict the occurrence of neonatal bacteremia. Despite the large number of hospitalized neonates, the incidence of bacteremia is low, ranging from one to 170 per 1000 live births [3,4]. Studies have shown that C-reactive protein (CRP) levels begin to rise 10-12 hours after the onset of infection. Therefore, CRP levels measured in delayed hours after birth are more predictive of neonatal infections compared to those measured immediately after birth [5,6].

Research suggests that in neonates with respiratory distress and minimal risk factors, transient tachypnea of the newborn (TTN) is often the primary cause, and observation alone may suffice without empirical antibiotic therapy [7,8]. However, even in such cases, basic blood tests, chest X-rays, and serial CRP testing conducted after admission are still performed to rule out other conditions such as pneumonia, persistent pulmonary hypertension of the newborn, or other diseases [7]. Consequently, most symptomatic neonates, regardless of antibiotic administration, undergo at least two rounds of blood tests.

Neonates possess delicate and small blood vessels, making blood sampling both technically challenging and frequently painful. Additionally, the procedure carries risks such as infection, blood loss, and considerable pain for the newborn [9,10]. Excessive or unnecessary phlebotomy can result in iatrogenic anemia due to notable hemoglobin depletion [11]. With the current trend toward minimizing empirical antibiotic use, reducing unnecessary blood tests in neonates has emerged as a crucial concern. Limiting the frequency of phlebotomy not only alleviates neonatal pain and reduces hemoglobin loss but also decreases the workload and time demands placed on healthcare providers.

This study aims to evaluate whether phlebotomy in hospitalized TTN neonates can be postponed after birth and whether a single blood draw can predict the association with neonatal bacteremia. This strategy seeks to reduce unnecessary blood tests, alleviate neonatal pain, mitigate the risk of hemoglobin depletion, and improve clinical efficiency.

## Materials and methods

Study designs and subjects

This study is a retrospective analysis conducted at E-Da Hospital, Kaohsiung, Taiwan. Data collection spanned from January 2021 to June 30, 2022. The study protocol received approval from the E-Da Hospital Institutional Review Board (approval number: EMRP33111N). To ensure patient confidentiality, all data were de-identified prior to statistical analysis.

At the outset, neonates born at E-Da Hospital and admitted to the neonatal ward were included in the study. Cases were subsequently filtered based on specific exclusion criteria. Medical records of these neonates were meticulously reviewed and documented, including baseline characteristics (e.g., gestational age, gender, mode of delivery, and birth weight), laboratory results obtained during hospitalization (e.g., complete blood count and biochemical analysis), the reports of blood bacterial cultures, and the medical histories and laboratory results of the neonates' mothers.

Examination protocol for hospitalized neonates at our institution

At our hospital, a standardized protocol is followed for the evaluation and blood testing of hospitalized neonates. Upon admission, a chest X-ray is routinely performed to rule out pneumothorax, meconium aspiration, or other lung parenchymal diseases such as pneumonia. For all neonates requiring hospitalization, two blood tests are arranged. The first blood test is conducted immediately after admission and includes a complete blood count with differential, blood culture, liver and renal function tests, and the first CRP measurement. The second blood test is performed 12-24 hours after birth, including assessments of neonatal electrolytes and the second CRP measurement.

Definition of TTN

TTN was defined as a diagnosis of exclusion in neonates presenting with respiratory distress or desaturation within the first hours of life, in combination with chest X-ray features (such as prominent central vascular markings, interlobar fissure fluid, and hyperinflation). When the attending neonatologist, after excluding other possible causes, considered TTN as the most likely diagnosis, the final diagnosis of TTN was documented in the discharge summary.

Definition of clinically significant bacteremia and CRP level

For cases with positive blood culture results, we defined clinically significant bacteremia as instances where subsequent treatment was initiated following the positive culture report. Cases with positive blood culture results but no further medical interventions, along with those where a repeat blood culture performed immediately after receiving a positive preliminary report returned negative, were excluded from the bacteremia group. Furthermore, the normal range for CRP is <5 mg/L in our hospital.

Statistical analysis

Statistical analyses were performed using IBM SPSS Statistics for Windows, Version 20.0 (IBM Corp., Armonk, New York, United States). Categorical variables were summarized as proportions, while continuous variables were presented as means±standard deviations. Group comparisons of continuous variables were conducted using the independent samples t-test, and the chi-squared test was applied for categorical variables. To adjust for potential confounders, multivariate logistic regression was employed to identify risk factors associated with bacteremia.

A random forest classifier was used to evaluate the relative importance of clinical biomarkers in predicting bacteremia. Two distinct random forest models were developed. The first model utilized only the first and second CRP levels as predictors, while the second model incorporated a broader range of clinical variables. Both models were trained to classify bacteremia, and feature importance was assessed using the mean decrease in impurity (Gini importance) to quantify the relative contribution of each predictor. In a random forest classifier, the feature importance score tells us how much each variable contributes to making accurate predictions. A higher importance score means the feature plays a bigger role in helping the model distinguish between the classes (e.g., predicting bacteremia vs. no bacteremia). A lower importance score means the feature contributes less and might not be as useful for prediction. All analyses were performed using Python, Version 3.9 (Python Software Foundation, Wilmington, Delaware, United States).

To evaluate the relative predictive value of the first and second measurements of CRP for blood culture positivity, we performed penalized logistic regression using the least absolute shrinkage and selection operator (LASSO). Both the first and the second CRP were entered as predictors, and fivefold cross-validation was applied to optimize the penalty parameter. Standardized coefficients were used to assess the importance of each variable.

## Results

From January 2021 to June 30, 2022, we collected data from a total of 559 cases, along with the corresponding maternal information. We excluded cases with congenital abnormalities (cyanotic heart disease (n=8; 1.43%); genetic abnormalities (n=3; 0.54%)), neonates with unstable vital signs after birth (those intubated immediately after birth or requiring inotropic agents during hospitalization (n=29; 5.19%)), meconium aspiration syndrome (n=5; 0.89%), pneumonia (n=3; 0.54%), persistent pulmonary hypertension of the newborn (n=5; 0.89%), pneumothorax (n=2; 0.36%), incomplete neonatal medical records (n=25; 4.47%), maternal cultures from genital secretions yielding confirmed bacterial infections before delivery (n=4; 0.72%), and maternal fever during labor (n=8; 1.43%), and cases where maternal antenatal care was conducted at another hospital or the maternal medical records were incomplete (n=61; 10.91%). After these exclusions, a total of 406 (72.63%) neonates diagnosed with TTN were included in this study.

Among the included cases, 10 neonates (2.46%) had positive blood cultures. The mean gestational age was 37.8±2.38 weeks, and the average birth weight was 2935.88±600.08 grams. Of the neonates, 237 (58.37%) were delivered vaginally, and 168 (41.38%) were female. Laboratory values for the neonates showed a mean white blood cell (WBC) count of 15523.03±5268.6 (/uL), a mean platelet count of 282495.07±63080.38 (/uL), and an immature-to-total neutrophil (I/T, %) ratio of 0.05±0.06. Regarding CRP, the first CRP was 0.41±2.67 mg/L, while the second CRP was 4.9±8.13 mg/L. Among the mothers, 15 (3.69%) experienced premature rupture of membranes (PROM) ≧18 hours. Maternal WBC and platelet counts were 10260.74±3613.87 (/uL) and 236867±62819.84 (/uL), respectively (Table 1). Among these bacteremia-positive cases, the identified pathogens were as follows: coagulase-negative *Staphylococcus* (n=4), *Enterococcus faecalis* (n=1), *Streptococcus agalactiae* (group B) (n=1), *Escherichia coli* (n=2), and *Micrococcus luteus* (n=2).

**Table 1:** Baseline characteristics of the enrolled subjects Continuous variables are presented as mean±standard deviation. Categorical variables are presented as number and percentage, n (%).

Neonatal factors	
Gestational age (weeks)	37.8±2.38
Birth weight (g)	2935.88±600.08
White blood cells (/uL)	15523.03±5268.6
Hemoglobin (g/dL)	17.16±1.96
Platelet (/uL)	282495.07±63080.38
Neutrophil (%)	56.27±13.21
Immature-to-total neutrophil ratio	0.05±0.06
C-reactive protein (first, mg/L)	0.41±2.67
C-reactive protein (second, mg/L)	4.9±8.13
Finger sugar	71.46±25.18
Apgar score (at one minute)	7.55±1.26
Apgar score (at five minutes)	8.72±1.01
Gender	
Female	168 (41.38%)
Male	238 (58.62%)
Mode of delivery	
Vaginal delivery	237 (58.37%)
Cesarean section	169 (41.63%)
Small for gestational age	
No	366 (90.15%)
Yes	40 (9.85%)
Blood culture	
No	396 (97.54%)
Yes	10 (2.46%)
Maternal factors	
White blood cells (/uL)	10260.74±3613.87
Platelet (/uL)	236867±62819.84
Premature rupture of membrane ≧18 hours	
No	391 (96.31%)
Yes	15 (3.69%)

Comparison of differences between neonates with and without positive blood cultures

When comparing neonates with and without bacteremia, factors such as gestational age, birth weight, WBC count, platelet count, antibiotic use, and CRP levels (first and second) were analyzed, as well as maternal factors such as WBC count, platelet count, and PROM ≧18 hours. Although no significant differences were observed, there was a trend toward higher WBC counts and second CRP levels in the bacteremia group (WBC: 15449.52±5216.50 vs. 18434±6720.42 (p=0.08); second CRP: 4.58±7.31 vs. 17.59±21.17 (p=0.08)) (Table 2). 

**Table 2:** Comparison of differences between neonates with and without bacteremia Continuous variables were analyzed using the independent samples t-test. Categorical variables were analyzed using the chi-squared test.

	Non-bacteremia (n=396) (97.54%)	Bacteremia (n=10) (2.46%)	t-value/chi-squared value	P-value
Neonatal factor				
Gestational age (weeks)	37.82±2.29	36.81±4.91	0.65	0.53
Birth weight (grams)	2941.27±590.69	2722.50±915.96	0.75	0.47
White blood cells (/uL)	15449.52±5216.50	18434±6720.42	-1.77	0.08
Hemoglobin (g/dL)	17.17±1.96	16.87±2.11	0.48	0.63
Platelet (/uL)	282601.01±63117.63	278300±64768.05	0.21	0.83
Neutrophil (%)	56.27±13.27	56.12±11.43	0.04	0.97
Immature-to-total neutrophil ratio	0.05±0.06	0.09±0.09	-1.15	0.28
C-reactive protein (first, mg/L)	0.30±1.72	4.39±13.18	-0.98	0.35
C-reactive protein (second, mg/L)	4.58±7.31	17.59±21.17	-1.94	0.08
Finger sugar	71.54±25.27	68.60±22.17	0.36	0.72
Apgar score (one minute)	7.55±1.27	7.60±0.84	-0.13	0.9
Apgar score (five minutes)	8.72±1.01	8.70±0.67	0.05	0.96
Gender				
Female	165 (41.67%)	3 (30%)	0.17	0.68
Male	231 (58.33%)	7 (70%)		
Mode of delivery				
Vaginal delivery	228 (57.58%)	9 (90%)	2.99	0.08
Cesarean section	168 (42.42%)	1 (10%)		
Small for gestational age				
No	358 (90.4%)	8 (80%)	0.31	0.58
Yes	38 (9.6%)	2 (20%)		
Antibiotic use				
No	35 (8.84%)	2 (20%)	1.47	0.23
Yes	361 (91.16%)	8 (80%)		
Maternal factor				
White blood cells (/uL)	10246.97±3566.29	10806±5400.62	-0.48	0.63
Platelet (/uL)	236845.96±62764.77	237700±68465.81	-0.04	0.97
Premature rupture of membrane ≧18 hours				
No	382 (96.46%)	9 (90%)	0.05	0.83
Yes	14 (3.54%)	1 (10%)		

Logistic regression analysis for determining factors associated with bacteremia

To identify factors associated with bacteremia, logistic regression analysis was performed using variables commonly related to infection. Univariate regression revealed that both the first and second CRP were significantly associated with bacteremia. Multivariate logistic regression showed that the second CRP was the only independent predictor of bacteremia (adjusted odds ratio 1.09; 95% CI 1.03-1.15; p<0.05) (Table 3). 

**Table 3:** Logistic regression analysis for determining factors associated with bacteremia Crude and adjusted odds ratios (Exp(B)) with 95% confidence intervals (CI) were calculated using binary logistic regression analysis. Variables with p-values <0.05 were considered statistically significant.

	Crude		Adjusted	
Variable	Exp(B) (95% CI)	p	Exp(B) (95% CI)	p
Gestational age	0.89 (0.74-1.06)	0.2	0.85 (0.52-1.4)	0.53
Birth weight	1.0 (1.0-1.0)	0.26	1.0 (1.0-1.0)	0.58
Mode of delivery	0.15 (0.02-1.2)	0.07	0.01 (0.0-1.91)	0.09
White blood cell	1.0 (1.0-1.0)	0.08	1.0 (1.0-1.0)	0.35
Immature-to-total neutrophil ratio	138.75 (0.33-58237.52)	0.11	13.87 (0.0-142738.08)	0.58
First C-reactive protein	1.12 (1.03-1.23)	<0.05	1.1 (0.94-1.27)	0.23
Second C-reactive protein	1.08 (1.04-1.12)	<0.05	1.09 (1.03-1.15)	<0.05
Finger sugar	1.0 (0.97-1.02)	0.72	0.98 (0.95-1.02)	0.31
Premature rupture of membrane ≧18 hours	3.03 (0.36-25.61)	0.31	1.84 (0.04-84.09)	0.75

Random forest classifier to evaluate the relative importance of various clinical biomarkers in predicting bacteremia

**Figure 1:**
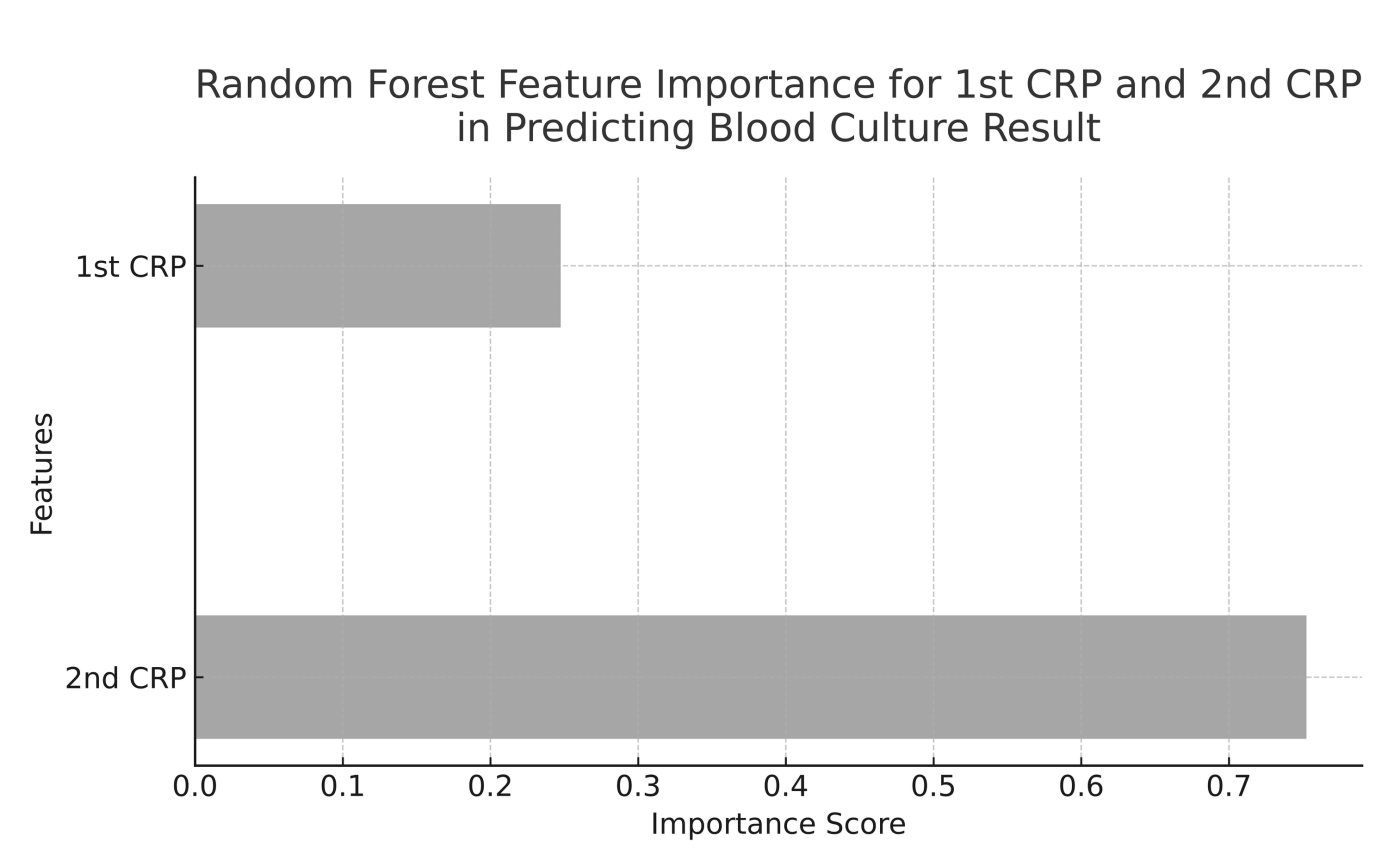
The importance of the first CRP and second CRP in predicting blood culture results Feature importance analysis from the random forest model. The second measurement of CRP shows the highest predictive value, while the first measurement contributes significantly less. CRP: C-reactive protein

Random forest analysis was used to assess the importance of clinical biomarkers in predicting bacteremia. If we compare only the first CRP and the second CRP, the results showed that the most significant biomarker was the second CRP, with an importance score of 0.7524, indicating its strong contribution to predicting positive blood cultures. The first CRP had a lower importance score of 0.2476 (Figure 1). 

**Figure 2:**
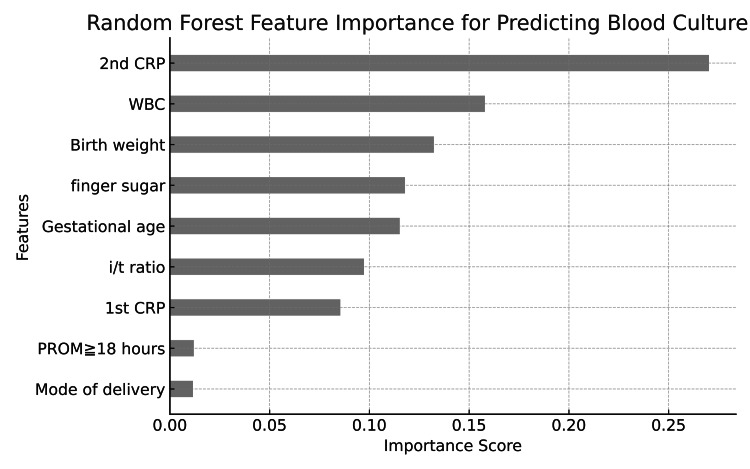
Feature importance analysis from the random forest model including multiple clinical variables The second measurement of CRP remains the most significant predictor, followed by WBC count and birth weight. CRP: C-reactive protein; WBC: white blood cell; i/t: immature-to-total neutrophil; PROM: premature rupture of membranes

Using a broader set of factors, the random forest analysis confirmed that the second CRP was still the most important predictor, with an importance score of 0.2702, followed by WBC and birth weight. Mode of delivery and PROM ≧18 hours had the lowest importance scores of 0.0116 and 0.0120, respectively. These findings reinforce the pivotal role of the second CRP in predicting bacteremia, with the first CRP showing relatively less impact (Figure 2). 

Assessment of the predictive importance of the first and second CRP measurements for bacteremia using LASSO regression

**Figure 3:**
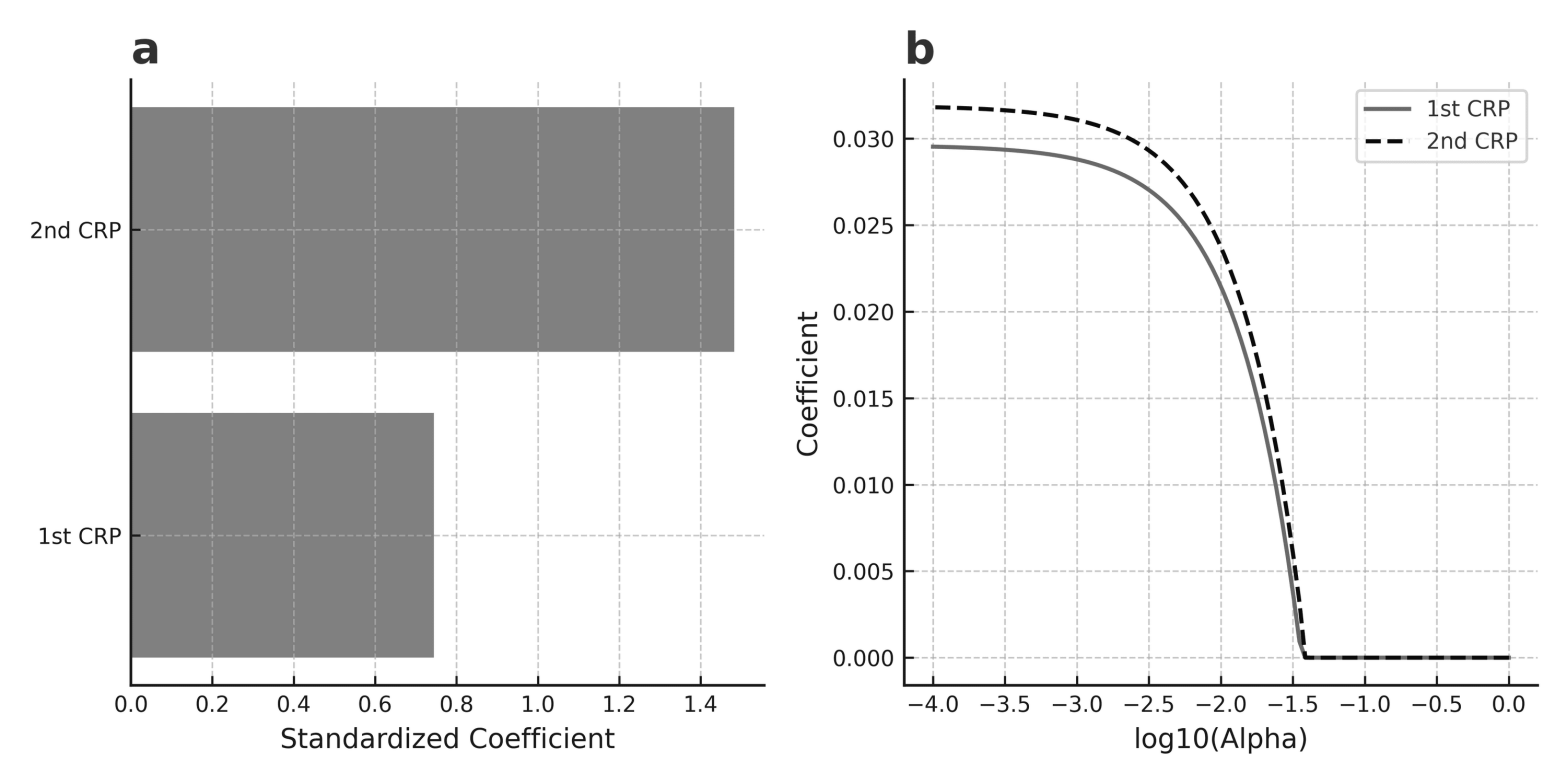
LASSO regression analysis of the first and second CRP for predicting blood culture positivity (a) Standardized coefficients of the first CRP and second CRP obtained from a LASSO logistic regression model. The larger coefficient of the second CRP indicates higher predictive importance for blood culture positivity after penalization. (b) LASSO coefficient path plots for the first CRP and second CRP. The second CRP measurement entered the model earlier and maintained a larger coefficient across a range of penalty strengths, supporting its greater contribution to the predictive model. CRP: C-reactive protein; LASSO: least absolute shrinkage and selection operator

In the LASSO model, the second CRP exhibited a higher standardized coefficient (+1.48) compared to the first CRP (+0.74), suggesting greater predictive importance after penalization. The coefficient path plot showed that the second CRP entered the model earlier and maintained a larger coefficient across penalty levels (Figure 3). 

## Discussion

Bacterial sepsis remains a significant contributor to morbidity and mortality among infants in neonatal intensive care units [3]. Diagnosing neonatal sepsis is particularly challenging, especially during its early stages. Neonatal infections often present with non-specific clinical findings such as poor digestion, reduced activity, or frequent apnea [12,13]. Despite ongoing efforts to identify reliable parameters for predicting neonatal sepsis, the challenge persists. Commonly used clinical laboratory data, such as CRP and the immature-to-total neutrophil ratio, have been widely studied, as have inflammatory cytokines (e.g., interleukin-1β, interleukin-6, interleukin-8, tumor necrosis factor-α, and the soluble receptor of interleukin-2). Additionally, heart rate-based parameters have proven to be reliable components in clinical decision support algorithms. Emerging approaches, including gene expression signatures and machine learning models, have also been explored for the early prediction of neonatal sepsis [5,14-18].

When neonatal sepsis is suspected, blood cultures remain the gold standard for its diagnosis [19]. While awaiting the final blood culture report, even if a neonate is hospitalized due to TTN without clear infection risk factors, it is common practice to initiate empirical antibiotic therapy in all newborns presenting with respiratory distress to avoid delaying treatment for early-onset sepsis [8,20]. As a result, many neonates receive unnecessary antibiotic therapy, which can contribute to the development of antibiotic resistance and alter the neonatal microbiota [21,22]. Similarly, neonates hospitalized for non-specific symptoms often undergo multiple serial blood samplings. Neonates in the intensive care unit experience an average of 7.5-17.3 painful procedures daily, with a higher frequency observed in those born at lower gestational ages [23]. Among these procedures, heel lance and venepuncture account for a significant proportion [23]. Painful exposures during the neonatal period have been associated with altered brain development and reduced neurodevelopmental outcomes in childhood. They may also result in activity-driven changes in pain pathways [24,25]. Moreover, excessive and unnecessary blood sampling frequently leads to neonatal anemia [26]. Therefore, reducing the frequency and volume of unnecessary blood draws is a critical issue that warrants active intervention.

Even in cases of early-onset sepsis in neonates, the sensitivity of CRP measurements immediately after birth is known to be unreliable [6]. This raises an important question: Is it truly necessary to perform blood tests immediately after hospitalization? Our study results indicate that late CRP measurement was identified as the sole significant predictor for bacteremia in the multivariate logistic regression analysis. The higher importance score of the second CRP in the random forest analysis further underscores its clinical relevance. In the random forest analysis, whether comparing the CRP levels measured 12-24 hours after birth with those measured immediately after birth or including other factors such as WBC counts and PROM, the late CRP consistently emerged as the most important predictor of bacteremia. From these findings, we can infer that for neonates with stable vital signs, normal activity, and no significant risk factors who are hospitalized due to respiratory distress or other non-specific symptoms, routine blood tests can be safely deferred to 12-24 hours after birth. This approach not only minimizes unnecessary pain for these neonates and reduces the risk of anemia caused by excessive blood draws but also alleviates the workload on healthcare providers. Furthermore, pediatricians can utilize this delayed CRP level to make more informed decisions regarding the initiation of antibiotic therapy, thereby reducing the unnecessary use of antibiotics in neonates.

Our study has several limitations. First, this is a retrospective study with data collected from a single medical center, and the sample size of neonates included in this study may not be large enough. Additionally, the study specifically focuses on the management process for neonates diagnosed with TTN. The neonates included in this analysis were diagnosed with TTN and were not all managed by the same physician. However, all neonates in our hospital are cared for by board-certified neonatologists, who determine the diagnosis of TTN based on comprehensive clinical presentations, laboratory data, and imaging studies, ensuring diagnostic accuracy. Furthermore, this study did not include other inflammatory markers, such as procalcitonin, for comparison. While some studies suggest that procalcitonin may have higher accuracy than CRP in predicting neonatal sepsis [27,28], others indicate that the comparison between procalcitonin and CRP remains controversial [29,30]. Additionally, procalcitonin is not yet widely used as a standard tool for diagnosing neonatal sepsis in most hospitals. Therefore, exploring the role of CRP in neonatal sepsis remains a meaningful and clinically relevant focus. Moreover, as our study focused on the relationship between late CRP checks and bacteremia rather than validating CRP as a diagnostic tool, diagnostic parameters such as sensitivity, specificity, and predictive values were not calculated. Given the small number of bacteremia cases (n=10) in our cohort, calculating these parameters would likely yield imprecise estimates. Additionally, as noted in the Introduction, the incidence of neonatal bacteremia ranges from approximately one to 170 per 1000 live births. Therefore, the absolute number of bacteremia cases in clinical practice is inherently low, which limits the statistical power and generalizability of studies in this field. Nevertheless, despite these limitations, we believe that our findings provide valuable insights into the potential role of late CRP measurements in identifying neonates at risk of bacteremia. This preliminary analysis highlights important considerations for clinical decision-making and serves as a foundation for future prospective studies with larger multicenter cohorts.

## Conclusions

In this retrospective study of neonates diagnosed with TTN, we found that CRP levels measured 12-24 hours after birth were significantly associated with neonatal bacteremia, whereas CRP levels measured immediately after admission were not. The second CRP not only emerged as an independent predictor of bacteremia in multivariate logistic regression but also demonstrated the highest predictive importance in random forest analysis. These findings indicate that, in clinically stable neonates with TTN and no evident infection risk factors, routine laboratory testing may be safely postponed. A single blood draw performed at 12-24 hours of life may be sufficient for sepsis evaluation and clinical decision-making. Implementing this approach may help reduce unnecessary phlebotomy, minimize procedural pain, lower the risk of iatrogenic anemia, and improve the efficiency of neonatal care. It may also reduce the overuse of empirical antibiotics and contribute to more targeted, patient-centered treatment strategies in neonatal medicine.
